# Individual-Based Simulation Models of HIV Transmission: Reporting Quality and Recommendations

**DOI:** 10.1371/journal.pone.0075624

**Published:** 2013-09-30

**Authors:** Nadia N. Abuelezam, Kathryn Rough, George R. Seage III

**Affiliations:** Department of Epidemiology, Harvard School of Public Health, Boston, Massachusetts, United States of America; Imperial College London, United Kingdom

## Abstract

**Background:**

Individual-based modeling is a growing technique in the HIV transmission and prevention literature, but insufficient attention has been paid to formally evaluate the quality of reporting in this field. We present reporting recommendations for individual-based models for HIV treatment and prevention, assess the quality of reporting in the existing literature, and comment on the contribution of this model type to HIV policy and prediction.

**Methods:**

We developed reporting recommendations for individual-based HIV transmission mathematical models, and through a systematic search, used them to evaluate the reporting in the existing literature. We identified papers that employed individual-based simulation models and were published in English prior to December 31, 2012. Articles were included if the models they employed simulated and tracked individuals, simulated HIV transmission between individuals in a particular population, and considered a particular treatment or prevention intervention. The papers were assessed with the reporting recommendations.

**Findings:**

Of 214 full text articles examined, 32 were included in the evaluation, representing 20 independent individual-based HIV treatment and prevention mathematical models. Manuscripts universally reported the objectives, context, and modeling conclusions in the context of the modeling assumptions and the model’s predictive capabilities, but the reporting of individual-based modeling methods, parameterization and calibration was variable. Six papers discussed the time step used and one discussed efforts to maintain internal validity in coding.

**Conclusion:**

Individual-based models represent detailed HIV transmission processes with the potential to contribute to inference and policy making for many different regions and populations. The rigor in reporting of assumptions, methods, and calibration of individual-based models focused on HIV transmission and prevention varies greatly. Higher standards for reporting of statistically rigorous calibration and model assumption testing need to be implemented to increase confidence in existing and future modeling results.

## Introduction

HIV transmission is influenced by numerous interactions between the biology of the virus and the behavior of individuals. The dynamics of transmission, treatment, and prevention are increasingly being represented by infectious disease mathematical models, which are accepted in the HIV literature as powerful predictive tools that motivate policy and inform clinical trial design [1]. Given the complexities of HIV transmission, models that represent individual-level behavior and partnering can be especially valuable. Individual-based simulations increase flexibility by allowing for heterogeneous individuals, interactions between individuals, correspondence to real life data, and a representation of the environment with which individuals interact [2,3]. This model type also allows individuals to have rationality in their actions (as opposed to completely random behavior) and simulates learning at individual and population levels [2]. However, individual-based models are difficult to parametrize, analyze, and generalize due to their complexity, leaving researchers to balance the advantages and disadvantages of this model type in the context of the problem of interest. As researchers begin large scale community randomized trials to assess the costs and benefits of HIV treatment and prevention interventions, there is a need for sophisticated and validated individual-level models to inform these studies’ designs.

The need for more consistent evaluation and comparison of mathematical models in the literature is a common theme in current modeling reviews and papers [4-6]. Individual-based model users have noted that there has been a decline in the reporting standards in the literature, and this may be contributing to its lack of use in many fields [4,7]. Previous reviews have aimed to provide the proper guidelines and documentation for mathematical models and simulations in the literature [5,6,8-11], but none have focused on the specific issues related to reporting of individual-based models in the context of HIV transmission and prevention.

In this paper, we describe the scope and quality of reporting of individual-based models in the HIV transmission and prevention literature. We first provide recommended reporting guidelines for individual-based mathematical models by tailoring previous mathematical modeling reporting guidelines to the individual-based model approach. We then apply these guidelines to existing HIV transmission and prevention individual-based models (found through a systematic search strategy) to assess the quality of reporting for this model type. We hope these guidelines will be a starting point for discussion with modelers to form standardized reporting guidelines for the united goal of improving the quality of the individual-based HIV modeling literature, and increasing their use among policy making consumers.

## Methods

### Reporting Recommendations

The following recommended reporting guidelines were constructed by expanding upon those presented in previous modeling reviews and from the experience of the authors (Table 1). The guidelines highlight the necessary components of general mathematical model reporting and the specific issues related to individual-based model reporting. We present each recommended guideline and provide the rationale for including the item with reference to the literature. Our guidelines do not rely on a specific reporting structure or article layout, as individual-based models are published in a wide variety of journal types. Instead our recommendations are organized into six sections according to different aspects of model development and presentation: 1) rationale, scope, and objective; 2) structure and features; 3) parameters; 4) assessment and validation; 5) presentation of results and conclusions; 6) authorship and funding (see Table 1).

**Table 1:** Recommended reporting guidelines for individual-based models of HIV transmission and prevention*.

**Topic**	**#**	**Item**							
***RATIONALE, SCOPE, AND OBJECTIVES***									
Title and Abstract	1	Identify in the title or abstract that the analysis depends on an **individual-based** mathematical model.							
Objective	2	State the objectives of the analysis with specific reference to the population(s), intervention(s), and time period(s) of interest.							
Context	3	Justify the exploration of the policy question in the context of previous trials, cohorts, and modeling analyses for the time period(s) of interest.							
Model Justification	4	**Explain the need for an individual-based model in the context of the objectives by referencing necessary model features.**							
***STRUCTURE AND FEATURES***									
Structure	5	Describe the model’s structure in both words and figures and describe how it affords the ability to explore the question(s) of interest.							
Assumptions	6	State the assumptions implicit in the model structure and justify with knowledge and data from the population of interest.							
Validity of Sexual Behavior	7	Justify the validity of the necessary behavior accounted for in the model.							
Validity of Biology	8	Justify the validity of the necessary biology accounted for in the model.							
***PARAMETERS***									
Parameters	9	List fixed parameters and calibrated parameters with ranges justified by the literature.							
Time step	10	**State and justify the length of the time step used to advance model dynamics, if applicable.**							
Heterogeneity	11	**Discuss how biological and behavioral heterogeneity is implemented in the model structure and whether this implementation allows for flexibility and specificity.**							
Interaction	12	Describe the parameters used to implement individual interaction in the model and justify the data used to parameterize these parameters.							
***ASSESSMENT AND VALIDATION***									
Calibration	13	**If the objective of the analysis is to describe or predict dynamics in a particular population of interest, describe the process used to calibrate the model dynamics to existing data including the statistical procedure, the types of outcome measures used, and the quality of the data used.**							
Sensitivity Analyses	14	Summarize the results of sensitivity analyses on the main model parameters, discuss whether the results support the robustness of findings, and describe future work needed.							
Assumption Sensitivity	15	Discuss how the behavior and inference of the model changes when particular assumptions (**e.g. alternative mixing patterns, different levels of heterogeneity for behavior and biology**) are altered or deleted.							
Stochastic Sensitivity	16	**Summarize the impact of stochasticity on the model runs and justify through random seed variation and sample size variation.**							
Internal Validity	17	**Describe the validity of the model programming by discussing how model bugs and program issues were checked and if modifications to model implementation were explored.**							
***PRESENTATION OF RESULTS AND DISCUSSION***									
Data Quality	18	Describe the quantity and quality of the data used to inform parameters for the population(s) of interest.							
Data Conversion	19	**Discuss issues related to the conversion of data to fit the time step used.**							
Results	20	Present key modeling results with uncertainty estimates and **indicate how many parameter sets were run for each analysis.**							
Limitations and Strengths	21	Provide the key limitations and strengths of the modeling study.							
Reproducibility	22	Discuss whether the model is able to reproduce the behavior of other populations or interventions of interest.							
Discussion	23	Interpret the modeling analysis within realistic bounds, with reference to previous modeling studies, a discussion about the generalizability of the modeling results, and implications for future studies or models.							
***AUTHORSHIP AND FUNDING***									
Authorship and Funding	24	List sources of funding and describe each author’s contribution to the modeling framework and conceptualization.							

* Bolded guidelines are specific to individual-based models of HIV transmission and prevention. Non-bolded guidelines are adaptable across different types of models.

#### Item 1: Title and Abstract – Identify in the title or abstract that the analysis depends on an individual-based mathematical model

Whether the structure of a mathematical model can be identified depends on how it is indexed in the literature, which relies on an informative title [12] and abstract. By indicating in the title or abstract that the analysis involves an individual-based mathematical model, authors alert readers to be aware of particular assumptions, structure, and details in the body of the paper.

#### Item 2: Objective – State the objectives of the analysis with specific reference to the population(s), intervention(s), and time period(s) of interest

Objectives should address the questions that the mathematical modeling exercise aims to answer, and reflect the efficacy, feasibility, and/or affordability of a particular HIV treatment or prevention intervention. A specific and focused purpose helps frame the paper’s methods and clarify the paper’s goals. The specificity of the objectives, especially with respect to the population, intervention, and time period of interest, justifies the model structure and indicates the data needs of the analysis. The information on setting and population will be essential for readers to assess the applicability and generalizability of the mathematical modeling results [12].

#### Item 3: Context – Justify the exploration of the policy question in the context of previous trials, cohorts, and modeling analyses for the time period(s) of interest

Authors should explain how their analysis adds to the existing literature by noting previous trials, studies, and modeling exercises that address similar questions. The political and social context of the tested intervention should also be discussed, to inform the readers of the potential consequences of this exploration [5]. The need for a new model should be justified if there is expansion on an existing model or model structure.

**Item 4: Model Justification – Explain the need for an individual-based mathematical model in the context of the objectives by referencing necessary model features (e.g. a need for heterogeneity of individual behavior and/or biology, and/or the explicit modeling of the interaction between individuals**)

The reasoning for the use of an individual-based model should be clarified early in the paper structure. Because our reporting guidelines are specific to mathematical analyses with a specific intervention, the need to justify a model’s design becomes necessary. Authors should emphasize the need for an individual-based model design by discussing the necessity of heterogeneity as well as explicit interaction of individuals in the context of the objectives, or other model necessary model features they have incorporated that are essential to their analysis [8,10,13]. . 

#### Item 5: Structure – Describe the model’s structure in both words and figures and describe how it affords the ability to explore the question(s) of interest

By indicating the model structure in both words and figures, the authors are able to communicate effectively with the readers about the capability of the model structure and the validity of the model’s assumptions [8]. Figures are important for those unfamiliar with mathematical modeling, as they give a visual representation of what is happening inside of the model structure. Avoiding the “black-box” phenomenon will allow readers to better judge the quality of mathematical models in the literature and the subsequent results that arise from them [14]. Without a clear understanding of the structure of a given mathematical model, readers will have a difficult time piecing together which analyses and explorations are feasible.

#### Item 6: Assumptions – State the assumptions implicit in the model structure and justify with knowledge and data from the population of interest

The generalizability of the conclusions drawn from the model analysis are dictated by the model’s assumptions [13]. Data used to justify assumptions should be included, so readers are aware of all limitations of the modeling approach. Simplifying assumptions related to the interaction of individuals in the population and the progression of HIV should be highlighted, as these have a direct impact on the authors’ ability to make accurate inferences.

#### Item 7: Validity of Sexual Behavior – Justify the validity of the necessary behavior accounted for in the model

Many aspects of sexual behavior are important to the transmission and prevention of HIV in a population, and the level of detail inherent in individual-based models with respect to sexual behavior is much higher than any other type of modeling [15]. Authors should discuss all relevant behavioral processes including, but not limited to: relationship types, relationship durations, directionality in men who have sex with men (MSM) partnerships, number of sex acts per time step, presence or absence of risk groups, mixing pattern, mechanism of acquisition of partnerships, age of sexual debut, change in sexual behavior with aging or time, presence or absence of migration, and the presence or absence of testing and the treatment cascade. The elements of sexual behavior included in the model will depend on the analysis and population of interest and should be justified using data and information from the population of interest [8]. Authors should acknowledge poorly understood behaviors and limitations in data used to inform parameters.

#### Item 8: Validity of Biology - Justify the validity of the necessary biology accounted for in the model

The level of biological detail that an individual-based model should represent is dependent upon the aims of the analysis of interest. Many elements of individual- and community-level biology are important to the transmission and prevention of HIV, for example: the inclusion or exclusion of sexually transmitted infection, the tracking of virological markers (CD4/HVL), multiple HIV disease stages with differing transmission probabilities, impact of circumcision status on HIV transmission, presence or absence of opportunistic infections, the impact of treatment on health and future transmission events, and the presence or absence of resistance mutations. By describing the elements incorporated in the model, the readers are able to determine which aspects of HIV transmission and prevention can be assessed.

#### Item 9: Parameters - List fixed parameters and calibrated parameters with ranges justified by the literature

When individual-based models begin to represent sexual behavior and biology, the number of parameters needed to populate them grows dramatically [10]. By listing the main parameters examined in the analyses of interest, the readers are able to understand the necessary sources of the data. Further, including uncertainty ranges establishes the need for attaining parameter values through a fitting or calibration procedure (see Item 13) when data is not available from the literature. Where parameter values cannot be based on the literature or are not calibrated, data from similar populations or assumptions made about the population of interest should be provided [7,8].

#### Item 10: Time Step - State and justify the length of the time step used to advance model dynamics, if applicable

The time step in an individual-based model is determined by the level of detail desired and computational limitations [13]. A short time step will provide greater detail on sexual behavior and biology, but will cause longer run times. Explicitly stating the time step, where appropriate, will make clear which processes the model describes well and makes the necessary data conversions for parameters transparent (see Item 19).

#### Item 11: Heterogeneity - Discuss how biological and behavioral heterogeneity is implemented in the model structure and whether this implementation allows for flexibility and specificity

One of the main strengths of the individual-based model structure is the ability to represent heterogeneity in behavior and biology [3] and, as such, methods used to implement this heterogeneity need to be detailed. Specific details on the discrete categories or continuous distributions used should be reported in the manuscript or supplementary material. Emphasizing the particular elements of behavior and biology that vary across individuals allows readers to understand how accurately these processes reflect reality and highlights strengths of the modeling exercise.

#### Item 12: Interaction - Describe the parameters used to implement individual interaction in the model and justify the data used to parameterize these parameters

HIV transmission that occurs within a particular partnership needs to be modeled accurately. Partnership formation and dissolution, the number of sexual acts in each partnership, the directionality of MSM partnerships, and the types of partnerships should be described in detail. By putting emphasis on the interaction between individuals in an individual-based model, authors will clearly describe the level of interaction between agents and consequently justify the need for this interaction.

**Item 13: Calibration - If the objective of the analysis is to describe or predict dynamics in a particular population of interest, describe the process used to calibrate the model dynamics to existing data including the statistical procedure, the types of outcome measures used, and the quality of the data used**

The process through which the model’s predictions, with regard to particular outcomes, are matched to data in the population of interest is called calibration [9]. There are many methods through which calibration can be statistically rigorous [5,9] and other methods through which it can be performed less rigorously. Calibration may be the most important piece of reported information for inferring a model’s ability to make accurate inferences about a particular population; therefore, details on the algorithms used and the outcomes calibrated should be provided. Although a model only needs to be calibrated once to reflect dynamics in a particular population, the calibration process should be repeated if the population, time period, or outcome of interest change. Models that have previously been calibrated should cite relevant previous manuscripts, and briefly describe the process. Authors should also make note of any effort to avoid over-fitting the model to data [16].

**Item 14: Sensitivity Analyses - Summarize the results of sensitivity analyses on the main model parameters, discuss whether the results support the robustness of findings, and describe future work needed**

As with any statistical analysis, it is essential to understand the sensitivity of the results to perturbations in parameters that are directly related to the intervention of interest [7]. For example, a model investigating how vaccine coverage impacts HIV incidence should vary vaccine coverage and efficacy to understand how model structure and parameterization impact the results. Sensitivity analyses may identify potential areas of model improvement and these discoveries should be noted. Less important results can be reported in supplemental material or an appendix.

**Item 15: Assumption Sensitivity - Discuss how the behavior and inference of the model changes when particular assumptions (e.g. alternative mixing patterns, different levels of heterogeneity for behavior and biology**)** are altered or deleted**

After describing the assumptions inherent in the model structure (see Item 6), the authors should discuss how the model’s behavior was dependent on these assumptions [8]. Assumption sensitivity analyses will reinforce the necessity of individual-based model structure and highlight which aspects of the intervention other model types would not capture. This type of sensitivity analysis will allow the authors to explore the generalizability of the model results to situations where the assumptions are violated [7,9]. Modelers will usually alter the assumptions most relevant for the analysis being performed, but altering other assumptions will improve the plausibility of the model results. Assumption sensitivity analyses can be performed in many different ways, one of which compares the results from the individual-based model to a deterministic model with simplified dynamics.

#### Item 16: Stochastic Sensitivity - Summarize the impact of stochasticity on the model runs and justify through random seed variation and sample size variation

Microsimulation models are often stochastic, meaning there is a certain level of randomness inherent to each model run [9,13]. Stochasticity can affect large simulation models in different ways, depending on which processes rely on random number generation. Authors should describe how stochasticity affects model results and what the authors have done to understand these effects, including increasing sample size or seeding the population differently [7].

#### Item 17: Internal Validity - Describe the validity of the model programming by discussing how model bugs and program issues were checked and if modifications to model implementation were explored

The internal validity of the model should be discussed, highlighting the steps taken to debug the model program and check the validity of the model structure [7,10]. This information should be placed in an appendix or supplemental material. Publications on previously published models should refer back to the methods of the original manuscript, but should not necessarily repeat the internal validity checks, unless the program code was changed. It is good practice to have two individuals program the model independently and compare the implementation to avoid bugs [7], though we acknowledge limited personnel or resources may make this infeasible. Model flowcharts, debugging runs, and other measures can also prevent bugs in the implementation of the code.

#### Item 18: Data Quality - Describe the quantity and quality of the data used to inform parameters for the population(s) of interest

As mentioned in Item 9, the number of parameters needed to populate an individual-based model is large, and it may be difficult to find data for the parameters needed. A discussion of the data quality for the population and region of interest will allow readers to understand the limitations to modeling in this population [10] and may encourage public health researchers and social scientists to collect additional relevant data.

#### Item 19: Data Conversion - Discuss issues related to the conversion of data to fit the time step used

The time step used is not often motivated by the data available, but rather by the goal of realism set by the authors. Data often has to be converted to the proper time step (e.g. number of sexual acts per partnership per time step). Any additional assumptions required for conversion should be discussed.

#### Item 20: Results - Present key modeling results with uncertainty estimates and indicate how many parameter sets were run for each analysis

Authors should report their results with uncertainty estimates. Particularly for individual-based models, authors should disclose the number of parameter sets used or runs averaged to get the results. Discussing uncertainty in results will help the authors anchor their conclusions (see Item 23) and give the readers a better understanding of the capabilities of the model.

#### Item 21: Limitations and Strengths - Provide the key limitations and strengths of the modeling study

The strengths and limitations of the methods used should be highlighted [12]. The limitations of an individual-based model are often dependent on the assumptions made, the computational power available, and the data used to inform the parameters. Detailed models of sexual behavior and transmission should emphasize the strengths of these details, while recognizing the potential weaknesses in data used to inform these processes.

#### Item 22: Reproducibility - Discuss whether the model is able to reproduce the behavior of other populations or interventions of interest

The generalizability of results (discussed in Item 23) should be emphasized along with the generalizability of the model structure. Some models are flexible enough to describe the behavior of many populations, while others are best suited to the dynamics in a single population. Describing whether the software and programming needed for implementing the model structure can be used to answer other questions of interest should be noted. A description of the generalizability of the model may encourage collaboration with other modeling groups or authors who have interest in using the model structure to answer alternative questions.

**Item 23: Discussion – Interpret the modeling analysis within realistic bounds, with reference to previous modeling studies, a discussion about the generalizability of the modeling results, and implications for future studies or models**

The discussion section of a modeling paper should emphasize the capability of the model to represent real world dynamics, while keeping the conclusions grounded upon the model assumptions. The generalizability of the results should be discussed, with a particular focus on the assumptions that allow for generalizability of the findings [12]. Future modeling and non-modeling studies should be proposed with insight as to how this body of work would contribute to the HIV literature as a whole.

#### Item 24: Authorship and Funding - List sources of funding and describe each author’s contribution to the modeling framework and conceptualization

Listing of funding sources allows other modelers to better understand what types of funding sources are applicable to modeling projects and grants. Additionally, by listing all the authors’ contributions, additional modeling teams or task forces can be composed based on capabilities of authors on previous projects and analyses.

### Search

Following the development of these reporting guidelines, we systematically reviewed the current individual-based HIV transmission and prevention literature to better understand the quality of the reporting in this field and ways in which it can be improved.

We searched PubMed, EMBASE, BIOSYS, and Web of Science for modeling papers published in English prior to December 31, 2012. The search used the following terms modified to the particular search language of each of the databases: an HIV infection term to capture papers related to HIV, transmission and prevention terms to capture papers examining these particular interventions, and simulation terms to capture models. To avoid confusion over vocabulary or classification of the modeling papers, a broad number of search terms were used to capture individual-based simulation models. Details on search terms can be found in Text S1.

**Figure 1:**
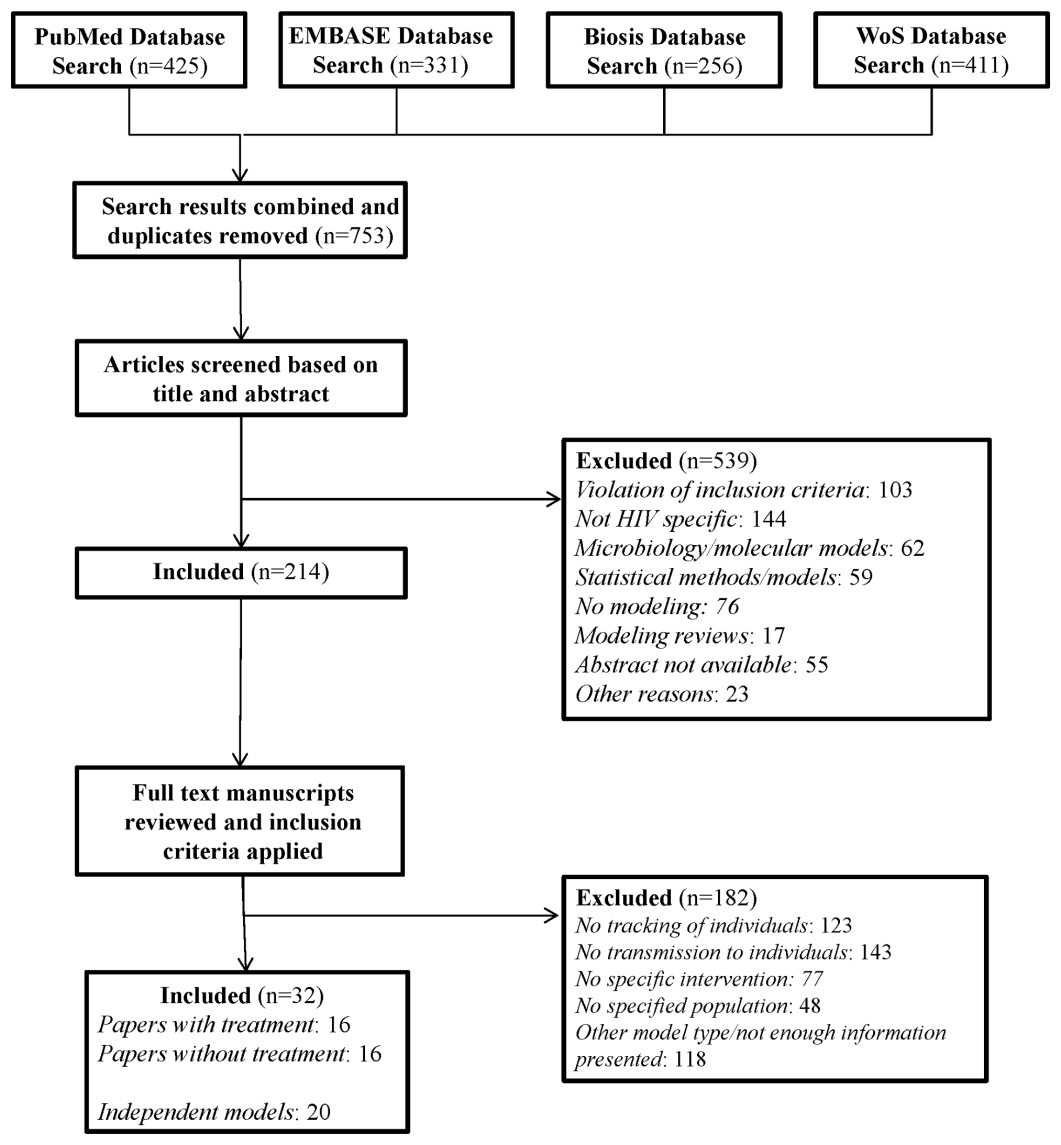
Cascade of papers excluded and included in the systematic review of individual-based HIV transmission, treatment, and prevention models in the literature.

Following the removal of duplicates across databases, all titles and abstracts were screened for exclusion. If the title and abstract did not provide enough information to evaluate the inclusion criteria or the information provided suggested the model was relevant, the full text was examined and evaluated based on the inclusion criteria. Two authors (NA and KR) conducted the search (Figure 1).

### Inclusion and Exclusion Criteria

Papers were included in the evaluation if they described a model that tracked individual characteristics and histories and simulated HIV transmission between specific individuals. They had to simulate a population denoted either geographically and/or behaviorally (e.g. men who have sex with men in the Southern United States) and examine the effects of a particular intervention (e.g. circumcision rollout). These inclusion criteria allowed us to capture models that simulated HIV transmission and prevention realistically, while eliminating models that were exploratory or theoretical in nature.

We excluded mathematical models that did not account for the interaction between individuals, as we believe that behavior is an essential element of individual-based models aiming to replicate HIV transmission. Papers that represented the probability of acquiring HIV using mass action equations or the proportion of individuals infected were not considered to model direct interaction. We also excluded modeling reviews, conference abstracts, and unpublished studies, as they did not provide enough information on model structure to warrant evaluation.

Multiple papers utilizing the same model to answer different questions of interest were included and evaluated, acknowledging that details of model structure could be included in previously published papers. For each paper identified in the systematic review, the quality of reporting was evaluated based on the described recommendations. Assessment of the included models was independently undertaken by two authors (NA and KR).

## Results

### Characteristics of Studies

The search criteria identified 1,423 citations, of which 753 were unique records (Figure 1). After initial screening of abstracts and titles, 214 citations were reviewed in full text, and 32 citations were included in the systematic review and evaluation. The characteristics of the included studies are summarized in Table 2 **.**

**Table 2:** Information on the 32 papers describing individual-based HIV treatment models that passed the inclusion criteria.

**Authors**	**Year**	**Journal**	**Model Name**	**Population of Interest**	**Treatment Modeled?**	**Intervention**
Adams et al. [17]	1998	Simulation	HIVSIM	Men who have sex with men (MSM) or heterosexual adults	No	Vaccine
Amirfar et al. [18]	2006	JAIDS		15-25 year old South African adolescent and adult females and their infants	Yes	Vaccine/PMTCT
Atkinson, J [48].	1996	Comput Biomed Res		Injection drug users (IDU)	No	Behavioral
Bendavid et al. [37]	2010	Arch Intern Med		Heterosexual adult South Africans	Yes	Testing/Treatment
Bernstein et al. [25]	1998	Interfaces	SimulAIDS	Heterosexual adults in a generic east African city	No	Behavioral/STD testing
Beyrer et al. [26]	2012	Lancet		MSM residing in high (urban USA), middle (urban Peru), and low income countries	Yes	Behavioral
Bracher et al. [27]	2004	Stud Fam Plann		Heterosexual adults in rural southern Malawi	No	Behavioral
Enns and Brandeau [33]	2011	Health Care Manag Sci		Heterosexual Tanzanian adults	Yes	Behavioral
Freeman et al. [40]	2009	Vaccine	STDSIM	Heterosexual adults in Sub-Saharan Africa	No	STD vaccine
Gray et al. [19]	2003	AIDS	Gray	Heterosexual adult discordant Ugandan couples	Yes	Treatment/Vaccine
Gray et al. [23]	2007	AIDS	Gray	Heterosexual adult discordant Ugandan couples	No	Male circumcision
Gray et al. [20]	2011	Vaccine		MSM in NSW, Australia	Yes	Vaccine
Hallett et al. [39]	2011	PLoS Med		Stable HIV-1 serodiscordant heterosexual couples in South Africa	Yes	PrEP
Hallett et al. [28]	2011	Sex Transm Infect		MSM in the Netherlands	Yes	Behavioral/Testing
Hoare et al. [36]	2012	Sex Health		MSM in Victoria, Australia	Yes	Testing
Hontelez et al. [21]	2011	Vaccine	STDSIM	Heterosexual adults in a rural South African setting	Yes	Vaccine
Hontelez et al. [38]	2012	AIDS	STDSIM	Heterosexual adults in Sub-Saharan Africa	Yes	Treatment
Korenromp et al. [41]	2000	AIDS	STDSIM	Heterosexual adults in Mwanza, Tanzania	No	STD treatment
Korenromp et al. [45]	2002	AIDS	STDSIM	Heterosexual adults in Rakai, Uganda	No	STD treatment
Korenromp et al. [43]	2005	J Infect Dis	STDSIM	Heterosexual adults in Rakai and Masaka, Uganda and Mwanza, Tanzania	No	STD treatment
Marshall et al. [15]	2012	PLoS One		IDU/NIDU/MSM in the New York metropolitical statistical area	Yes	Behavioral/Testing
McCabe et al. [46]	2010	PLoS One		Pregnant women and infants in the United States	Yes	PMTCT
McCreesh et al. [34]	2011	Sex Transm Infect		Heterosexual adults in rural South-West Uganda	Yes	Behavioral
Rauner et al. [47]	2005	*J* *Oper* Res Soc		Pregnant women and infants in Tanzania	Yes	PMTCT
Robinson et al. [29]	1995	AIDS	SimulAIDS	Heterosexual adults in rural south-west Uganda	No	Behavioral/STD treatment
Van der Ploeg et al. [30]	1998	Interfaces	STDSIM	Heterosexual adults in Nairobi, Kenya	No	Behavioral/STD treatment
van Vliet et al. [31]	2001	Bull World Health Organ	STDSIM	Heterosexual adults	No	Behavioral
Vieira et al. [22]	2010	Ann. Oper Res		Urban heterosexual, MSM, and bisexual adult Brazilians	Yes	Treatment/Vaccine/Behavioral
Vissers et al. [32]	2011	Epidemiol Infect	STDSIM	Heterosexual adults in Tanzania	No	Behavioral
White et al. [44]	2004	JAIDS	STDSIM	Heterosexual adults in Rakai and Masaka, Uganda and Mwanza, Tanzania	No	STD treatment
White et al. [24]	2008	AIDS	STDSIM	Heterosexual adults in Sub-Saharan Africa	No	Male circumcision
Wilson et al. [35]	2011	Sex Transm Infect		MSM in Melbourne, Australia	No	Testing

Individual-based microsimulation models were first published in the 1980’s, though the oldest model to fit our inclusion criteria was published in 1995. The included analyses were published in a number of different journals (Table 2), ranging from computationally focused to medical journals. About half of them (n=15) were published in journals that had high eigenfactor and article influence (>90th percentile respectively). More than half of the studies (n=22) explored HIV transmission in heterosexual populations, eight studies analyzed HIV epidemics among MSM, and two studies focused on injection drug users (IDUs). Most studies (n=21) were populated with data from African countries. The interventions of interest in these models varied, with many examining the presence of a vaccine (n=6), behavioral interventions (n=13), and HIV testing and/or antiretroviral treatment (n=8) with several analyses featuring multiple interventions (n=9).

### Evaluation

**Figure 2:**
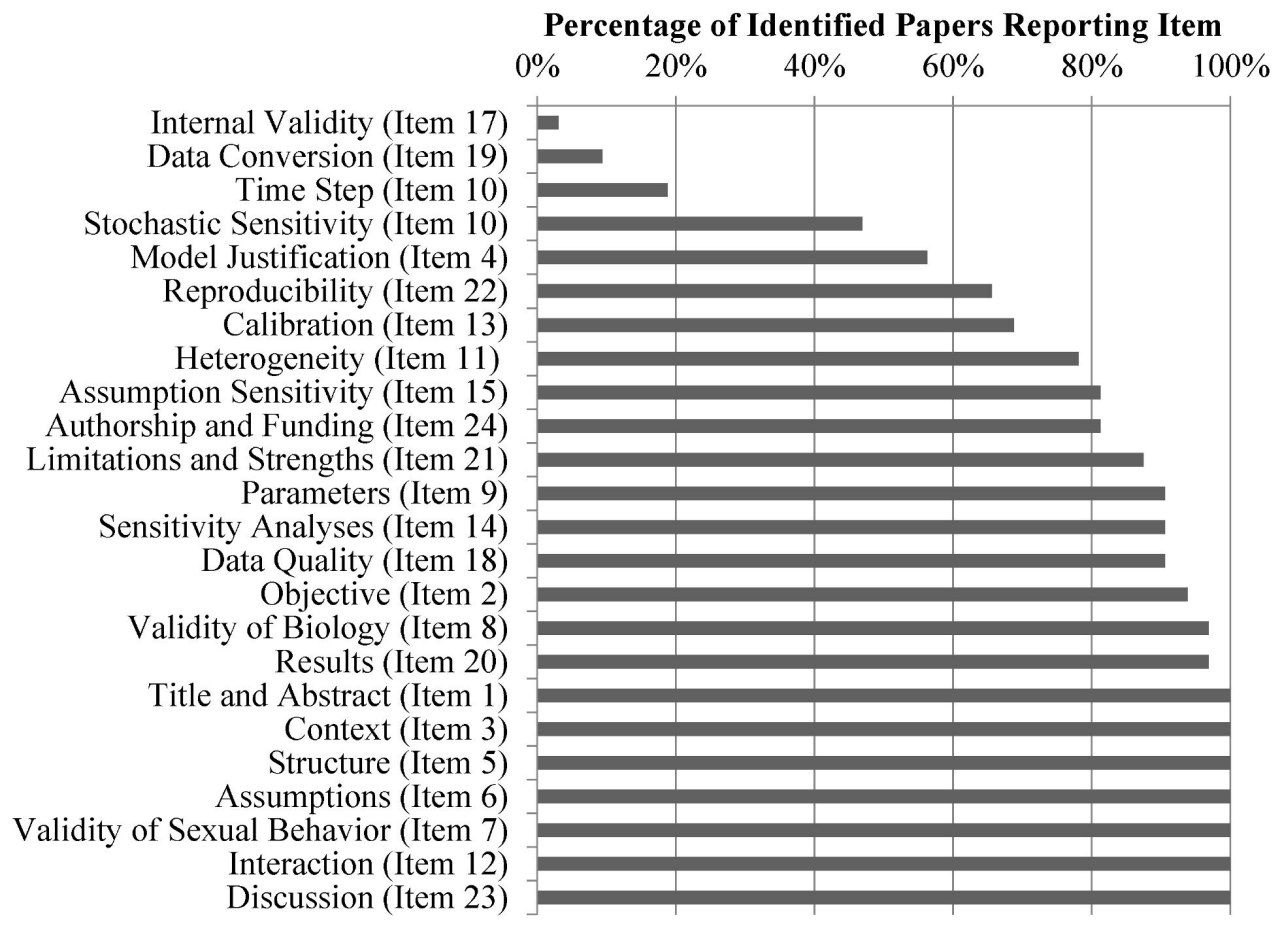
Bar chart of the percentage of identified papers that complied with each reporting guideline item.

The number and percentage of papers that complied with the recommended reporting guidelines are reported in Table 3 and Figure 2. Table S1 provides the details of this evaluation for each paper.

**Table 3:** Evaluation of reporting quality in individual-based models in the HIV transmission and prevention literature (N=32).

**Item**	**Total Number of Papers**
	**n (%)**
1. Title and Abstract	32 (100.0)
2. Objective	30 (93.8)
3. Context	32 (100.0)
4. Model Justification	18 (56.3)
5. Structure	32 (100.0)
6. Assumptions	32 (100.0)
7. Validity of Sexual Behavior	32 (100.0)
8. Validity of Biology	31 (96.9)
9. Parameters	29 (90.6)
10. Time step	6 (18.8)
11. Heterogeneity	25 (78.1)
12. Interaction	32 (100.0)
13. Calibration	22 (68.8)
14. Sensitivity Analyses	29 (90.6)
15. Assumption Sensitivity	26 (81.3)
16. Stochastic Sensitivity	15 (46.9)
17. Internal Validity	1 (3.1)
18. Data Quality	29 (90.6)
19. Data Conversion	3 (9.4)
20. Results	31 (96.9)
21. Limitations and Strengths	28 (87.5)
22. Reproducibility	21 (65.6)
23. Discussion	32 (100.0)
24. Authorship and Funding	26 (81.3)

The modeling papers almost universally described the context in which the analyses were performed and the objectives of the simulation studies. More than half of the papers (n=17) were not distinguished in the title as a mathematical model or a simulation analysis, but all papers did note the use of a model in the abstract. Fifty six percent of the papers (n=18) justified the use of an individual-based model as the necessary method to answer the question of interest. Nine of the models included a graphical representation of the model structure, while the rest described the structure in plain language. Some of the papers (n=9) referred to an appendix or a previous paper for more detail on the model structure. Nineteen percent (n=6) state and justify the length of the time step used and 78% of the papers (n=25) describe how heterogeneity is implemented in the model structure or recognize that heterogeneity was used in the individual-based modeling framework. Sixty nine percent (n=22) describe the process through which the model was calibrated to data, although six of the reviewed papers did not aim to accurately represent the dynamics in a population, but rather understand general trends, and so would not be expected to perform a calibration procedure. Nearly half (n=15) of the papers summarize the impact of stochasticity on the model results and discuss the magnitude of stochasticity in the model behavior. Sensitivity analyses describing how the model behaved when assumptions were altered or deleted were performed for 81% of the papers (n=26). One paper described the validity of the model programming, debugging procedure, and other details related to the implementation and validation of the programming. Three papers discussed the issue of converting data to fit the time step restrictions in the model structure. Nearly all papers (n=31) presented the modeling results clearly; eleven of these papers presented uncertainty estimates around their effect estimates and predicted values. All of the papers provided interpretations of the modeling results within realistic bounds without overinflating the usefulness of the results.

## Discussion

We found that individual-based models in the HIV transmission and prevention literature are able to answer a wide range of questions related to specific populations and interventions (Table 2). The models examined how a variety of HIV interventions such as vaccination [17-22], circumcision [23,24], condom usage [25-32], reduction in concurrency [25,26,29,32-34], HIV testing [15,28,35-37], anti-retroviral treatment [19,22,37-39], STD control [25,29,30,40-45], and prevention of mother to child transmission [18,46,47] can affect HIV incidence and prevalence in a wide variety of settings including North America [15,26,46], Australia [20,35,36] and sub-Saharan Africa [18,19,21,23-25,27,29,30,32-34,37-45,47]. These analyses were able to discuss the effects of interventions in less researched and accessible populations like MSM [15,17,20,22,26,28,35,36] and IDU [15,48].

We found that the reporting of results from individual-based model analyses was very strong with respect to the basics of public health research and other model analyses (e.g. stating the objective, giving context from the literature, and providing grounded conclusions) but lacking in the description of methods particular to individual-based models. Authors may feel uncomfortable giving detailed descriptions of the methods in a paper aimed at a general public health audience because the technical details may make the paper harder to read or understand. However, detailed reporting is essential to ensure that the quality of the literature remains high and the results are reproducible. To this extent, emphasis on reporting items related to structure (Item 5), assumptions (Item 6), calibration (Item 13) and strengths and limitations (Item 21) will be most important for individuals trying to understand published individual-based models. The more frequently modeling methods are included in public health analyses, the more widely the methods will be accepted and valued. Additionally, providing detail and transparency in methods will encourage collaboration among mathematical modelers, while making individual-based modeling more accessible to those unfamiliar with the process.

As evidenced by the diversity of journals the models were published in (Table 2), individual-based models are valued for their ability to represent existing epidemics for a wide variety of populations and regions. Just over half of the articles included in this review noted that the analysis relied on mathematical modeling in the title, which suggests that authors might feel that denoting a study as a modeling study could deter readers. However, as this practice becomes more common, modeling papers will be read with the same clarity and readiness as other types of analyses. As more journals recognize the utility of modeling, distinguishing model analyses from classic public health analyses becomes more important.

Individual-based models are used widely in other fields, including ecology [49], meteorology [50], and traffic monitoring [51], and it is encouraging to see their influence growing in the HIV literature. Individual-based modeling is user-friendly and highly visual, allowing for collaboration and understanding across multidisciplinary teams [52]. Strengths of individual-based models include their ability to model the interaction between individuals with great detail and to reflect heterogeneity in behavior and biology. Many authors noted the inability to completely parametrize their models from the literature, tractably analyze models without uncertainty, and the need for sophisticated methods of calibration to help increase their confidence in their findings. Deterrents to individual-based modeling in the HIV transmission literature include the lack of biological and behavioral data in many populations, which prevents parameterization of this complex model type, as noted in some reviewed papers [26,39].

The creation of individual-based models is a complex and arduous process, yet standards for reporting them are relatively non-existent. We aimed to provide guidelines to strengthen the reporting of results in this field and an overview of individual-based models examining HIV-related interventions. The existing literature is broad and thorough; however, more information is needed on the rigor of calibration and the rationale for the use of individual-based modeling. Future work in this field should aim to make the literature accessible to a general audience by using clear language that non-modelers and non-mathematicians can understand. The clearer the presentation, the more widely modeling literature will be read and applied in the future. By collaborating with other interested parties or modeling groups, we hope to develop a consensus statement on the reporting of individual-based models in the HIV treatment and prevention literature.

## Supporting Information

Text S1 **Search terms for systematic review.**
(DOCX)

Table S1 **Tabled evaluation of all eligible systematically identified individual-based HIV transmission models based on the reporting recommendations.**
(DOCX)
