# Supplementary material for: Individual-Based Simulation Models of HIV Transmission: Reporting Quality and Recommendations
Source: PLoS One. 2013 Sep 30;8(9):e75624. doi: 10.1371/journal.pone.0075624 (PMC3787035; doi:10.1371/journal.pone.0075624)
Supplement: Text S1 — Search terms for systematic review. (DOCX) [file pone.0075624.s001.docx]

**Text S1**: Search terms for systematic review

*PubMed Search Terms*

("HIV Infections"[Mesh] OR HIV[tiab] OR human immunodeficiency virus[tiab] OR LAV[tiab] OR HTLV III[tiab] OR T Lymphotropic virus Type III[tiab] OR AIDS[tiab] OR acquired immunodeficiency[tiab]) AND ("Disease Transmission, Infectious"[Mesh] OR "HIV Infections/transmission"[Mesh] OR "HIV Infections/prevention and control"[Mesh] OR transmission[tiab] OR prevention[tiab] OR preventive[tiab] OR prophylaxis[tiab]) AND ("Computer Simulation"[Mesh] OR microsimulation[tiab] OR simulation[tiab] OR agent based[tiab] OR individual-based[tiab] OR computer model*[tiab] OR computerized model*[tiab] OR in silico[tiab])

*EMBASE Search Terms*

('Human immunodeficiency virus infection'/exp OR ‘HIV’:ab,ti OR ‘human immunodeficiency virus’:ab,ti OR ‘LAV’:ab,ti OR ‘HTLV III’:ab,ti OR ‘T Lymphotropic virus Type III’:ab,ti OR ‘AIDS’:ab,ti OR ‘acquired immunodeficiency’:ab,ti) AND

('disease transmission'/de OR 'virus transmission'/de OR 'sexual transmission'/de OR 'vertical transmission'/de OR 'infection prevention'/de OR transmission:ab,ti OR prevention:ab,ti OR preventive:ab,ti OR prophylaxis:ab,ti) AND

('computer simulation'/de OR microsimulation:ab,ti OR simulation:ab,ti OR ‘agent based’:ab,ti OR ‘individual-based’:ab,ti OR (computer NEAR/1 model*):ab,ti OR (computerized NEAR/1model*):ab,ti OR ‘in silico’:ab,ti)

*BIOSYS and Web of Science Search Terms*

microsimulation OR simulation OR “agent based “ OR “individual-based“ OR computer* NEAR/1 model* OR “in silico“

AND

HIV OR “human immunodeficiency virus “ OR “LAV “ OR “HTLV III “ OR “T Lymphotropic virus Type III “ OR “AIDS “ OR “acquired immunodeficiency “

AND

transmission OR prevention OR preventive OR prophylaxis
