# Supplementary material for: Individual-Based Simulation Models of HIV Transmission: Reporting Quality and Recommendations
Source: PLoS One. 2013 Sep 30;8(9):e75624. doi: 10.1371/journal.pone.0075624 (PMC3787035; doi:10.1371/journal.pone.0075624)
Supplement: Table S1 — Tabled evaluation of all eligible systematically identified individual-based HIV transmission models based on the reporting recommendations. (DOCX) [file pone.0075624.s002.docx]

**Table S1:** Tabled evaluation of all eligible systematically identified individual-based HIV transmission models based on the reporting recommendations.

The following evaluation tables highlight the presence or absence of each of the criteria in the systematically identified modeling papers. The evaluations were done in a conservative manner, such that if a paper had one of the requirements within a particular criterion, it was considered satisfied. A “Yes” means the information was reported, while a “No” indicates that the information was not found in the paper by the evaluators.

**Table S1:** Evaluation of all eligible systematically identified individual-based HIV transmission models based on the reporting recommendations.

| **Guideline Topic and Number** | **Adams et al. 1998** | **Amirfar et al. 2006** | **Atkinson 1996** | **Bendavid et al. 2010** | **Bernstein et al. 1998** |
| --- | --- | --- | --- | --- | --- |
| *Rationale, Scope and Objectives* |  |  |  |  |  |
| 1 - Title and Abstract | Yes | Yes | Yes | Yes | Yes |
| 2 - Objective | No | Yes | No | Yes | Yes |
| 3 - Context | Yes | Yes | Yes | Yes | Yes |
| 4 - Model Justification | Yes | No | Yes | No | Yes |
| *Structure and Features* |  |  |  |  |  |
| 5 - Structure | Yes | Yes | Yes | Yes | Yes |
| 6 - Assumptions | Yes | Yes | Yes | Yes | Yes |
| 7 - Validity of Sexual Behavior | Yes | Yes | Yes | Yes | Yes |
| 8 - Validity of Biology | Yes | Yes | Yes | Yes | Yes |
| *Parameters* |  |  |  |  |  |
| 9 - Parameters | Yes | Yes | No | Yes | Yes |
| 10 - Time Step | No | No | No | Yes | No |
| 11 - Heterogeneity | Yes | No | No | Yes | Yes |
| 12 - Interaction | Yes | Yes | Yes | Yes | Yes |
| *Assessment and Validation* |  |  |  |  |  |
| 13 - Calibration | No | Yes | No | Yes | No |
| 14 - Sensitivity Analysis | No | Yes | Yes | Yes | Yes |
| 15 - Assumption Sensitivity | No | No | Yes | Yes | Yes |
| 16 - Stochastic Sensitivity | No | No | No | No | Yes |
| 17 - Internal Validity | No | No | No | No | No |
| *Presentation of Results and Discussion* |  |  |  |  |  |
| 18 - Data Quality | No | Yes | No | Yes | Yes |
| 19 - Data Conversion | No | No | No | No | No |
| 20 - Results | Yes | Yes | No | Yes | Yes |
| 21 - Limitations and Strengths | No | Yes | Yes | Yes | Yes |
| 22 - Reproducibility | No | Yes | Yes | No | No |
| 23 - Discussion | Yes | Yes | Yes | Yes | Yes |
| *Authorship and Funding* |  |  |  |  |  |
| 24 - Authorship and Funding | Yes | Yes | Yes | Yes | Yes |

**Table S1 (cont):** Evaluation of all eligible systematically identified individual-based HIV transmission models based on the reporting recommendations.

| **Guideline Topic and Number** | **Beyrer et al. 2012** | **Bracher et al. 2004** | **Enns et al. 2011** | **Freeman et al. 2009** | **Gray et al. 2003** |
| --- | --- | --- | --- | --- | --- |
| *Rationale, Scope and Objectives* |  |  |  |  |  |
| 1 - Title and Abstract | Yes | Yes | Yes | Yes | Yes |
| 2 - Objective | Yes | Yes | Yes | Yes | Yes |
| 3 - Context | Yes | Yes | Yes | Yes | Yes |
| 4 - Model Justification | Yes | Yes | Yes | Yes | No |
| *Structure and Features* |  |  |  |  |  |
| 5 - Structure | Yes | Yes | Yes | Yes | Yes |
| 6 - Assumptions | Yes | Yes | Yes | Yes | Yes |
| 7 - Validity of Sexual Behavior | Yes | Yes | Yes | Yes | Yes |
| 8 - Validity of Biology | No | Yes | Yes | Yes | Yes |
| *Parameters* |  |  |  |  |  |
| 9 - Parameters | Yes | Yes | Yes | Yes | Yes |
| 10 - Time Step | No | No | No | No | No |
| 11 - Heterogeneity | Yes | Yes | Yes | Yes | Yes |
| 12 - Interaction | Yes | Yes | Yes | Yes | Yes |
| *Assessment and Validation* |  |  |  |  |  |
| 13 - Calibration | No | Yes | Yes | Yes | No |
| 14 - Sensitivity Analysis | Yes | Yes | Yes | Yes | Yes |
| 15 - Assumption Sensitivity | Yes | Yes | Yes | Yes | Yes |
| 16 - Stochastic Sensitivity | Yes | No | Yes | No | No |
| 17 - Internal Validity | No | No | No | No | No |
| *Presentation of Results and Discussion* |  |  |  |  |  |
| 18 - Data Quality | Yes | Yes | Yes | Yes | Yes |
| 19 - Data Conversion | No | No | No | No | No |
| 20 - Results | Yes | Yes | Yes | Yes | Yes |
| 21 - Limitations and Strengths | No | Yes | Yes | Yes | Yes |
| 22 - Reproducibility | Yes | Yes | Yes | Yes | Yes |
| 23 - Discussion | Yes | Yes | Yes | Yes | Yes |
| *Authorship and Funding* |  |  |  |  |  |
| 24 - Authorship and Funding | Yes | Yes | No | Yes | Yes |

**Table S1 (cont):** Evaluation of all eligible systematically identified individual-based HIV transmission models based on the reporting recommendations.

| **Guideline Topic and Number** | **Gray et al. 2007** | **Gray et al. 2011** | **Hallett et al. 2011 (PLoS Med)** | **Hallet et al. 2011 (STI)** |
| --- | --- | --- | --- | --- |
| *Rationale, Scope and Objectives* |  |  |  |  |
| 1 - Title and Abstract | Yes | Yes | Yes | Yes |
| 2 - Objective | Yes | Yes | Yes | Yes |
| 3 - Context | Yes | Yes | Yes | Yes |
| 4 - Model Justification | No | Yes | No | No |
| *Structure and Features* |  |  |  |  |
| 5 - Structure | Yes | Yes | Yes | Yes |
| 6 - Assumptions | Yes | Yes | Yes | Yes |
| 7 - Validity of Sexual Behavior | Yes | Yes | Yes | Yes |
| 8 - Validity of Biology | Yes | Yes | Yes | Yes |
| *Parameters* |  |  |  |  |
| 9 - Parameters | Yes | Yes | Yes | Yes |
| 10 - Time Step | Yes | Yes | Yes | No |
| 11 - Heterogeneity | No | Yes | No | Yes |
| 12 - Interaction | Yes | Yes | Yes | Yes |
| *Assessment and Validation* |  |  |  |  |
| 13 - Calibration | No | Yes | No | No |
| 14 - Sensitivity Analysis | Yes | Yes | Yes | Yes |
| 15 – Assumption Sensitivity | Yes | No | Yes | Yes |
| 16 - Stochastic Sensitivity | No | Yes | Yes | No |
| 17 - Internal Validity | No | No | No | No |
| *Presentation of Results and Discussion* |  |  |  |  |
| 18 - Data Quality | Yes | Yes | Yes | Yes |
| 19 - Data Conversion | No | Yes | No | No |
| 20 - Results | Yes | Yes | Yes | Yes |
| 21 - Limitations and Strengths | Yes | Yes | Yes | Yes |
| 22 - Reproducibility | No | Yes | Yes | No |
| 23 - Discussion | Yes | Yes | Yes | Yes |
| *Authorship and Funding* |  |  |  |  |
| 24 - Authorship and Funding | No | Yes | Yes | Yes |

**Table S1 (cont):** Evaluation of all eligible systematically identified individual-based HIV transmission models based on the reporting recommendations.

| **Guideline Topic and Number** | **Hoare et al. 2012** | **Hontelez et al. 2011** | **Hontelez et al. 2012** | **Korenromp et al. 2000** | **Korenromp et al. 2002** |
| --- | --- | --- | --- | --- | --- |
| *Rationale, Scope and Objectives* |  |  |  |  |  |
| 1 - Title and Abstract | Yes | Yes | Yes | Yes | Yes |
| 2 - Objective | Yes | Yes | Yes | Yes | Yes |
| 3 - Context | Yes | Yes | Yes | Yes | Yes |
| 4 - Model Justification | No | Yes | No | Yes | Yes |
| *Structure and Features* |  |  |  |  |  |
| 5 - Structure | Yes | Yes | Yes | Yes | Yes |
| 6 - Assumptions | Yes | Yes | Yes | Yes | Yes |
| 7 - Validity of Sexual Behavior | Yes | Yes | Yes | Yes | Yes |
| 8 - Validity of Biology | Yes | Yes | Yes | Yes | Yes |
| *Parameters* |  |  |  |  |  |
| 9 - Parameters | Yes | Yes | Yes | Yes | Yes |
| 10 - Time Step | No | No | No | No | No |
| 11 - Heterogeneity | Yes | Yes | Yes | Yes | Yes |
| 12 - Interaction | Yes | Yes | Yes | Yes | Yes |
| *Assessment and Validation* |  |  |  |  |  |
| 13 - Calibration | Yes | Yes | Yes | Yes | Yes |
| 14 - Sensitivity Analysis | No | Yes | No | Yes | Yes |
| 15 - Assumption Sensitivity | Yes | No | Yes | Yes | Yes |
| 16 - Stochastic Sensitivity | Yes | Yes | No | No | No |
| 17 - Internal Validity | No | No | No | No | No |
| *Presentation of Results and Discussion* |  |  |  |  |  |
| 18 - Data Quality | Yes | Yes | Yes | Yes | Yes |
| 19 - Data Conversion | No | No | No | No | No |
| 20 - Results | Yes | Yes | Yes | Yes | Yes |
| 21 - Limitations and Strengths | Yes | Yes | Yes | Yes | Yes |
| 22 - Reproducibility | Yes | No | Yes | Yes | Yes |
| 23 - Discussion | Yes | Yes | Yes | Yes | Yes |
| *Authorship and Funding* |  |  |  |  |  |
| 24 - Authorship and Funding | No | No | Yes | Yes | Yes |

**Table S1 (cont):** Evaluation of all eligible systematically identified individual-based HIV transmission models based on the reporting recommendations.

| **Guideline Topic and Number** | **Korenromp et al. 2005** | **Marshall et al. 2012** | **McCabe et al. 2010** | **McCreesh et al. 2011** | **Rauner et al. 2005** |
| --- | --- | --- | --- | --- | --- |
| *Rationale, Scope and Objectives* |  |  |  |  |  |
| 1 - Title and Abstract | Yes | Yes | Yes | Yes | Yes |
| 2 - Objective | Yes | Yes | Yes | Yes | Yes |
| 3 - Context | Yes | Yes | Yes | Yes | Yes |
| 4 - Model Justification | Yes | Yes | No | Yes | Yes |
| *Structure and Features* |  |  |  |  |  |
| 5 - Structure | Yes | Yes | Yes | Yes | Yes |
| 6 - Assumptions | Yes | Yes | Yes | Yes | Yes |
| 7 - Validity of Sexual Behavior | Yes | Yes | Yes | Yes | Yes |
| 8 - Validity of Biology | Yes | Yes | Yes | Yes | Yes |
| *Parameters* |  |  |  |  |  |
| 9 - Parameters | No | Yes | Yes | Yes | No |
| 10 - Time Step | No | Yes | No | No | No |
| 11 - Heterogeneity | Yes | No | Yes | Yes | Yes |
| 12 - Interaction | Yes | Yes | Yes | Yes | Yes |
| *Assessment and Validation* |  |  |  |  |  |
| 13 - Calibration | Yes | Yes | Yes | Yes | Yes |
| 14 - Sensitivity Analysis | Yes | Yes | Yes | Yes | Yes |
| 15 - Assumption Sensitivity | Yes | No | Yes | Yes | Yes |
| 16 - Stochastic Sensitivity | Yes | Yes | No | Yes | Yes |
| 17 - Internal Validity | No | No | No | No | Yes |
| *Presentation of Results and Discussion* |  |  |  |  |  |
| 18 - Data Quality | Yes | Yes | Yes | Yes | Yes |
| 19 - Data Conversion | No | No | No | No | No |
| 20 - Results | Yes | Yes | Yes | Yes | Yes |
| 21 - Limitations and Strengths | Yes | Yes | Yes | Yes | Yes |
| 22 - Reproducibility | Yes | No | No | No | Yes |
| 23 - Discussion | Yes | Yes | Yes | Yes | Yes |
| *Authorship and Funding* |  |  |  |  |  |
| 24 - Authorship and Funding | Yes | Yes | Yes | No | Yes |

**Table S1 (cont):** Evaluation of all eligible systematically identified individual-based HIV transmission models based on the reporting recommendations.

| **Guideline Topic and Number** | **Robinson et al. 1995** | **Van der Ploeg et al. 1998** | **van Vliet et al. 2001** | **Vieira et al. 2010** | **Vissers et al. 2011** |
| --- | --- | --- | --- | --- | --- |
| *Rationale, Scope and Objectives* |  |  |  |  |  |
| 1 - Title and Abstract | Yes | Yes | Yes | Yes | Yes |
| 2 - Objective | Yes | Yes | Yes | Yes | Yes |
| 3 - Context | Yes | Yes | Yes | Yes | Yes |
| 4 - Model Justification | No | Yes | Yes | Yes | No |
| *Structure and Features* |  |  |  |  |  |
| 5 - Structure | Yes | Yes | Yes | Yes | Yes |
| 6 - Assumptions | Yes | Yes | Yes | Yes | Yes |
| 7 - Validity of Sexual Behavior | Yes | Yes | Yes | Yes | Yes |
| 8 - Validity of Biology | Yes | Yes | Yes | Yes | Yes |
| *Parameters* |  |  |  |  |  |
| 9 - Parameters | Yes | Yes | Yes | Yes | Yes |
| 10 - Time Step | No | No | No | Yes | No |
| 11 - Heterogeneity | No | Yes | Yes | Yes | Yes |
| 12 - Interaction | Yes | Yes | Yes | Yes | Yes |
| *Assessment and Validation* |  |  |  |  |  |
| 13 - Calibration | Yes | Yes | Yes | No | Yes |
| 14 - Sensitivity Analysis | Yes | Yes | Yes | Yes | Yes |
| 15 - Assumption Sensitivity | No | Yes | Yes | Yes | Yes |
| 16 - Stochastic Sensitivity | Yes | Yes | Yes | Yes | No |
| 17 - Internal Validity | No | No | No | No | No |
| *Presentation of Results and Discussion* |  |  |  |  |  |
| 18 - Data Quality | Yes | Yes | Yes | Yes | Yes |
| 19 - Data Conversion | No | Yes | No | Yes | No |
| 20 - Results | Yes | Yes | Yes | Yes | Yes |
| 21 - Limitations and Strengths | Yes | Yes | No | Yes | No |
| 22 - Reproducibility | No | Yes | Yes | No | Yes |
| 23 - Discussion | Yes | Yes | Yes | Yes | Yes |
| *Authorship and Funding* |  |  |  |  |  |
| 24 - Authorship and Funding | Yes | Yes | Yes | No | Yes |

**Table S1 (cont):** Evaluation of all eligible systematically identified individual-based HIV transmission models based on the reporting recommendations.

| **Guideline Topic and Number** | **White et al. 2004** | **White et al. 2008** | **Wilson et al. 2011** |
| --- | --- | --- | --- |
| *Rationale, Scope and Objectives* |  |  |  |
| 1 - Title and Abstract | Yes | Yes | Yes |
| 2 - Objective | Yes | Yes | Yes |
| 3 - Context | Yes | Yes | Yes |
| 4 - Model Justification | No | No | No |
| *Structure and Features* |  |  |  |
| 5 - Structure | Yes | Yes | Yes |
| 6 - Assumptions | Yes | Yes | Yes |
| 7 - Validity of Sexual Behavior | Yes | Yes | Yes |
| 8 - Validity of Biology | Yes | Yes | Yes |
| *Parameters* |  |  |  |
| 9 - Parameters | Yes | Yes | Yes |
| 10 - Time Step | No | No | No |
| 11 - Heterogeneity | No | Yes | Yes |
| 12 - Interaction | Yes | Yes | Yes |
| *Assessment and Validation* |  |  |  |
| 13 - Calibration | Yes | Yes | No |
| 14 - Sensitivity Analysis | Yes | Yes | Yes |
| 15 - Assumption Sensitivity | Yes | Yes | Yes |
| 16 - Stochastic Sensitivity | No | No | No |
| 17 - Internal Validity | No | No | No |
| *Presentation of Results and Discussion* |  |  |  |
| 18 - Data Quality | No | Yes | Yes |
| 19 - Data Conversion | No | No | No |
| 20 - Results | Yes | Yes | Yes |
| 21 - Limitations and Strengths | Yes | Yes | Yes |
| 22 - Reproducibility | Yes | Yes | Yes |
| 23 - Discussion | Yes | Yes | Yes |
| *Authorship and Funding* |  |  |  |
| 24 - Authorship and Funding | Yes | Yes | Yes |
